# Outcome of Tuberculosis Treatment in Patients with Diabetes Mellitus Treated in the Revised National Tuberculosis Control Programme in Malappuram District, Kerala, India

**DOI:** 10.1371/journal.pone.0076275

**Published:** 2013-10-14

**Authors:** Nandakumar KV, Karthickeyan Duraisamy, Shibu Balakrishnan, Sunilkumar M, Jaya Sankar S, Karuna D. Sagili, Srinath Satyanarayana, Ajay Kumar MV, Donald A. Enarson

**Affiliations:** 1 District Tuberculosis Centre, Manjeri, Malappuram District, Kerala, India; 2 Department of Tuberculosis, WHO Country Office for India, New Delhi, India; 3 State TB Training and Demonstration Centre, Trivandrum, Kerala, India; 4 State TB Cell, Trivandrum, Kerala, India; 5 International Union Against Tuberculosis and Lung Disease, South East Asia Regional Office, New Delhi, India; 6 International Union Against Tuberculosis and Lung Disease, Montreal, Quebec, Canada; Institute of Infectious Diseases and Molecular Medicine, South Africa

## Abstract

**Settings:**

Kerala State, India has reported the greatest dual burden of Tuberculosis (TB) and Diabetes Mellitus (DM). Malappuram district in Kerala has monitored and recorded DM status and its control from 2010 under Revised National Tuberculosis Control Program (RNTCP).

**Objectives:**

To assess, under programme conditions, comprehensiveness of recording DM status among TB cases and the TB treatment outcomes among DM patients (disaggregated by glycemic control) and compare with-non DM patients.

**Design:**

This retrospective record review included 3,116TB patients from April 2010 to September 2011.DM was defined as per international guidelines and TB treatment outcomes were categorized as favourable(cured and treatment completed) and unfavourable(death, default, failure and transfer out). Relative Risk (RR) and 95% confidence intervals(CI) were calculated to assess the risk of unfavourable outcomes.

**Results:**

DM status was recorded in 90% of TB cases and 667 (24%) had DM. 17% of DM patients and 23% of patients with unknown DM status had unfavourable outcomes but this difference was not statistically significant. Unadjusted RR for poor glycemic control or unknown control status for unfavourable outcome were (2.00; 95% CI 0.97–4.13) and (2.14; 95% CI 1.11–4.13).

**Conclusion:**

This study could not confirm an adverse association between DM or its control during treatment and the course of response to TB treatment.DM screening in TB cases and recording of DM care needs to be improved to enable more conclusive evidence.

## Introduction

There is a clear association between Diabetes Mellitus (DM) and Tuberculosis (TB) [1]–[6].Patients with both conditions have been reported to have an increased risk of adverse outcomes of TB treatment including failure and death during treatment and subsequent relapse[7]–[12]. Few studies have investigated the association of the course of TB treatment and its outcome with the DM control status in these patients. [13]–[15]

The Indian State of Kerala reports the highest prevalence of DM in TB patients (44%) [16]. Though there are studies from India about prevalence of diabetes among TB patients, there is only one study from India evaluating the course and presentation of Tuberculosis among diabetic TB cases, by Dr.Hemalatha et al. Malappuram district in Kerala has routinely recorded DM status and its control in the treatment cards used in the Revised National Tuberculosis Control Programme (RNTCP) programme since 2010. This provided us the opportunity to study the association of DM and its control and the response to treatment in all types of TB patients registered under RNTCP in Malappuram district, Kerala, India, from April 2010 to September 2011[17], [18]. Specific objectives of the study were to: 1) evaluate the completeness of records of DM and its control in consecutive adult TB patients enrolled on treatment; 2) determine the association of DM and its control with the course and outcome of TB treatment.

## Materials and Methods

### Ethics statement

Ethics approval received from Ethics Advisory Group of International Union Against Tuberculosis and Lung Disease and Institution Ethics Committee of MES Medical College, Perintalmanna, Malappuram district. Administrative approval from the State TB Cell, Kerala was obtained for conducting the study. Since this was a record review, we were not able to obtain informed consent from individual patients. The same was presented to ethics committees and a waiver of informed consent was obtained.

### Study design

Retrospective cohort study involving review of records routinely collected and maintained within RNTCP.

### Study setting

Malappuram, the most populated district from Kerala state in India with a population of 4.14 million, has implemented Government of India's RNTCP since 1998.TB patients are treated with Directly Observed Treatment, Short-course (DOTS) chemotherapy three times a week throughout the treatment period, according to international recommendations. At the end of the initial intensive phase (IP) of treatment, if the sputum smear was positive the IP was extended by an additional month [19].

Diagnostic and follow up sputum smear examination was done in quality assured designated microscopic centers in the district. The progress and outcome of treatment was reported from 128 peripheral health institutions in the district. Treatment Cards and Tuberculosis Registers were maintained at eight sub district units (Tuberculosis Units). In Malappuram district all TB cases under RNTCP have been screened for DM and their care monitored since April 2010 by blood glucose estimation using the calorimetric method/Auto analysers(semi or fully automatic) in laboratory conditions and the instruments were periodically calibrating and tests were performed by trained qualified lab technicians. In the field setting, all field staff were using calibrated digital Glucometers andstandard test strips and all were formally trained in using the digital glucometer.The blood glucose results were routinely recorded in the treatment cards. Following a local innovation in the district, diabetes care has been standardized [20]–[23].

### Study Population and sampling

We included treatment cards of all TB cases above the age of 14 years, registered under RNTCP in Malappuram District of Kerala state, India, from April 2010 to September 2011 excluding Transfer- in cases.

Sample size was calculated using the following assumptions: Confidence level of 95%, Power of 80%, ratio of exposed (Diabetic TB) to un-exposed (non-Diabetic TB) of 4, favourable treatment outcome for TB cases without diabetes of 85% and size of a difference to be detected was 5%.

### Data collection

During the data collection the sensitized RNTCP key staff brought all treatment cards and the TB registers pertaining to the study period to District TB Centre and the principal investigator with their help abstracted the required data in a pre-tested structured data collection form from the treatment cards and TB registers at District TB centre from November 2012 to January2013.Comprehensiveness of ascertainment of treatment cards was ensured by cross checking with the TB register.

Variables were defined as per programme guidelines. All patients were considered to have DM if they reported the disease or a history of intake of anti diabetic drugs, or had a blood glucose value of >126 mg for Fasting blood sugar(FBS) or >200 mg for Random blood sugar(RBS) or for postprandial blood sugar(PPBS) during diabetic screening while on TB treatment[22], [23]. Operationally, we defined those with DM with at least three blood sugar values done during treatment, at least one month apart with at least one test done during continuation phase of treatment were assessed as having ‘known diabetic control’ status. Those with all these blood sugar values within normal limit(100 mg for FBS and 140 mg for RBS or PPBS) were considered ‘under control’[22]–[24].

### Data entry, analysis and statistics

Data was double entered using EpiData entry software (Version 3.1, EpiData Association, Odense, Denmark).Data bases were validated and discrepancies resolved through referral to the original data collection forms after which the data base was finalized, and securely locked. The finalized database contained no personal identifiers and was used for statistical analysis using EpiData analysis software (Version 2.2.2.178). We used chi-square test for comparing proportions and *P* value of ≤0.05 was considered statistically significant. Relative risk and 95% confidence intervals were determined to measure associations. Those variables found statistically significant during bivariate analysis and had adequate numbers were entered into a multivariate regression model (log-binomial regression) to calculate adjusted relative risks and assess independent effects of each variable. Multivariate analysis was conducted using Stata software (Stata Corp. 2011. Stata Statistical Software: Release 12. College Station, TX: Stata Corp LP.).

## Results

Malappuram district, Kerala, India registered 4,203 Tuberculosis cases under RNTCP from April 2010 to September 2011.After excluding all Transfer-in cases and TB cases below 15 years of age 3,116 TB patients were included in the study. There were no missing records. Of the cases 1,001(32%) were females. Mean(SD) age was 46(17)years. 2,708 (87%) were new cases. Pulmonary cases comprised 2,239(72%). Of the new cases 1,416(52%) were sputum smear positive, 459 (17%) were pulmonary sputum smear negative cases and 833(31%) were extra pulmonary TB cases. Of the re-treatment cases178 (44%) were relapses, 91(22%) were treatment after failure, 42(10%) were treatment after default and 97(24%) were re-treatment others. Of the 2,239 pulmonary TB cases, 1,727(77%) were smear positive with 697(40%) 3+ positive, 323(19%) 2+ positive, 436(25%) 1+ positive and 271(16%) scanty positive.

Human immunodeficiency virus (HIV) status was known in 2,572 (83%) of which 36(1.4%) were HIV positive. Treatment was taken regularly with no missed doses in 2,940(94%) during the intensive phase and 3,002(96%) in the continuation phase of treatment.

**Figure 1:**
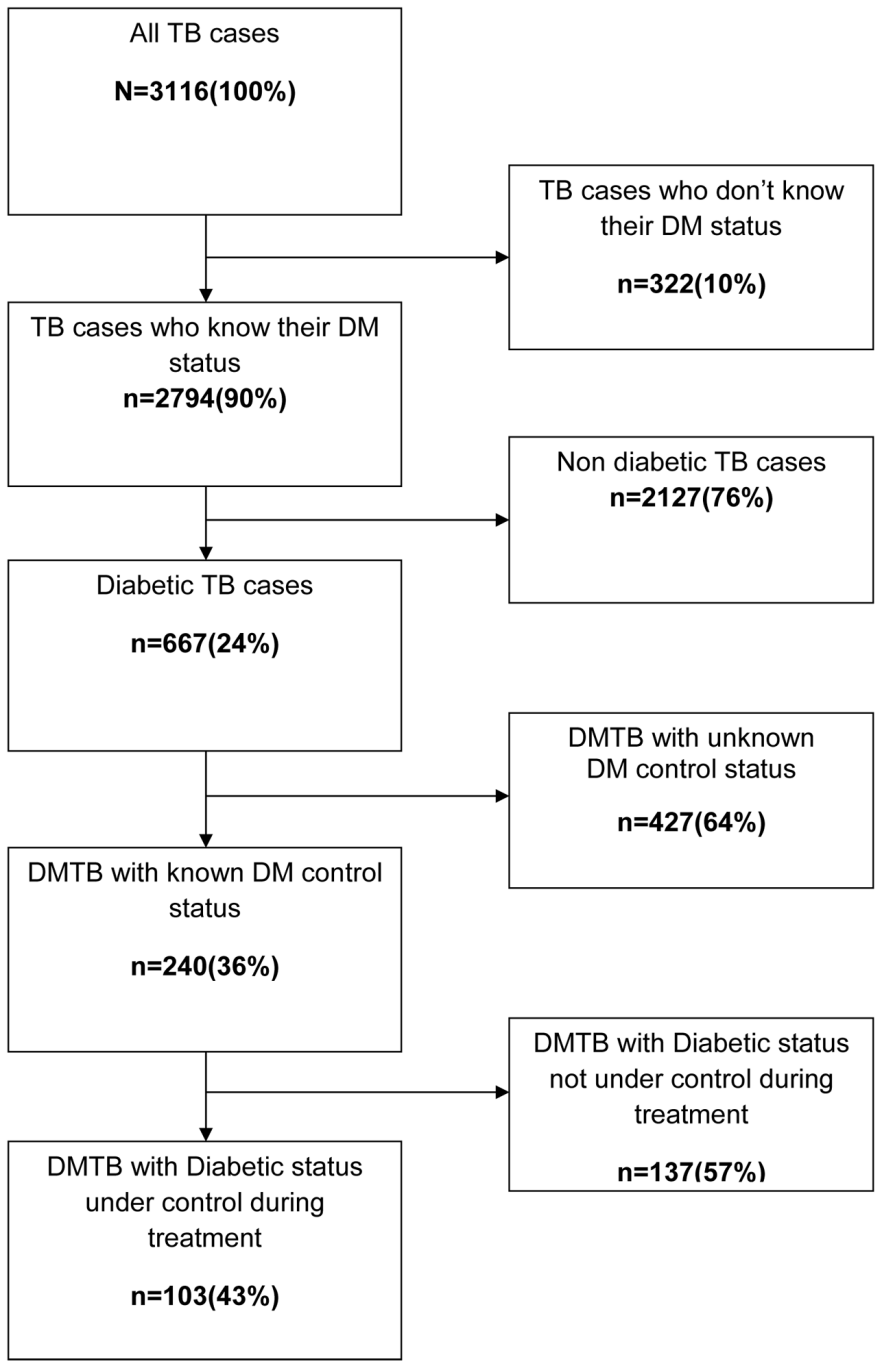
Tuberculosis patients with diabetes and diabetes control. Figure shows flow of Tuberculosis patients registered under the Revised National Tuberculosis Control Programme (RNTCP) in Malappuram District, Kerala, India, from April 2010 to September 2011, by diabetes and diabetes control status. Out of 3116 TB patients, 2794(90%) know their diabetic status and of this 667(24%) are diabetic. Out of the diabetic TB patients 240(36%) know their diabetic control status during treatment and of this 103(43%) are under diabetic control during treatment.

In 2,794 (90%) cases DM status was known of which 667(24%) had DM (figure 1). Of the DM patients, only240 (36%) cases had known diabetic control status during the treatment period of which 103(43%) cases were under diabetic control (representing only 15% of total DM TB cases). DM patients were more likely to be male, age above 45 years, have pulmonary tuberculosis, be retreatment cases and be sputum smear positive (table 1).

**Table 1:** Demographic and clinical characteristics of tuberculosis patients at the time of recording in the Revised National Tuberculosis Control Programme in Malappuram District, Kerala, India, April 2010 to September 2011, stratified by Diabetes Mellitus status (N = 3116).

		Diabetes mellitus status						
Characteristic		No		Yes		Unknown		
		No.	(%)	No.	(%)	No.	(%)	p-value
**Total**		2,127	(100)	667	(100)	322	(100)	
**Sex**								**<0.001**
	Female	757		141	(21)	103	(32)	
	Male	1,370		526	(79)	219	(68)	
**Age group**								**<0.001**
	15–44	1,057		128	(19)	148	(46)	
	45+	1,070		539	(81)	174	(54)	
**HIV Status**								**<0.001**
	Negative	1,830	(86)	581	(87)	125	(39)	
	Positive	27	(1)	5	(1)	4	(1)	
	Unknown	270	(13)	81	(12)	193	(60)	
**Site**								**<0.001**
	Pulmonary	1,454	(68)	565	(85)	220	(68)	
	Extra pulmonary	673	(32)	102	(15)	102	(32)	
**Type of case**								**<0.001**
	New	1,870	(88)	550	(83)	288	(89)	
	Re-treatment	257	(12)	117	(17)	34	(11)	
**Sputum smear**								**<0.001**
	Positive	1,097	(52)	469	(70)	161	(50)	
	Negative/Unknown	1,030	(48)	198	(30)	161	(50)	

HIV- Human immune deficiency virus.

Sputum smear conversion was unrelated to DM status or to DM control (table 2). Treatment outcome according to DM categories is shown in table 3. Male sex, older age, pulmonary TB, retreatment cases, sputum smear positivity and regularity of DOT in the intensive phase were significantly associated with TB treatment outcome. DM and unknown DM status were also significantly associated with TB treatment outcome as was unknown DM control status.

**Table 2:** Smear conversion at three months of treatment of sputum positive Tuberculosis patients in Malappuram district, Kerala state,India from April 2010 to September 2011, by status of Diabetes Mellitus (n = 1727).

			Smear conversion at three months						
Diabetes status			No		Yes		RR	(95% CI)	p-value
			Number	(%)	Number	(%)			
**Total**			341	(100)	1,386	(100)			
**Diabetes**									
	No		216	(20)	881	(80)	Reference		
	Yes		83	(18)	386	(82)	0.90	(0.71–1.13	0.36
	Unknown		42	(26)	119	(74)	1.32	(1.00–1.76)	0.06
**Diabetes control**									
	Yes		22	(32)	46	(68)	Reference		
	No		38	(37)	65	(63)	1.14	(0.74–1.75)	0.54
	Unknown		97	(33)	201	(67)	1.01	(0.69–1.47)	0.98

RR = relative risk; CI = confidence interval.

**Table 3:** Outcome of treatment of tuberculosis in the Revised National Tuberculosis Control Programme in Malappuram District, Kerala, India, April 2010 to September 2011, by diabetes mellitus, demographic and clinical characteristics.

Characteristic	Treatment outcome			Relative risk				
	All	Unfavourable		Crude		Adjusted		
		n	%		95%CI		95%CI	
**Total**	3,116	474	(100)					
**Sex**								
Female	1,001	93	(9)					
Male	2,115	381	(18)	1.94	**1.57–2.40**	1.60	**1.28–1.99**	
**Age group**								
15–44	1,333	126	(9)					
45+	1,782	348	(19)	2.06	**1.71–2.50**	1.72	**1.40–2.10**	
**HIV status**								
Negative	2,536	344	(14)					
Positive	36	8	(22)	1.64	0.88–3.04	1.93	**1.06–3.50**	
Unknown	544	122	(22)		**1.37–1.99**	1.51	**1.23–1.84**	
**Site**								
Extra pulmonary	877	88	(10)					
Pulmonary	2,239	386	(17)	1.72	**1.38–2.14**	1.30		0.99–1.72
**Type of case**								
New	2,708	380	(14)					
Re treatment	408	94	(23)	1.64	**1.34–2.01**	1.43		**1.18–1.75**
**Sputum smear status**								
Negative/unknown	1,597	172	(18)					
Positive	1,579	362	(12)	1.41	**1.19–1.68**	1.02		0.86–1.28
**DOT in intensive phase**								
Regular	2940	405	(14)					
Missed doses	176	69	(39)	2.85	**2.32–3.49**			
**Diabetes**								
No	2,127	288	(13)					
Yes	667	113	(17)	1.25	**1.02–1.53**	0.99		0.81–1.21
Unknown	322	73	(23)	1.67	**1.33–2.10**	1.34		**1.05–1.70**
**Diabetic control**								
Yes	103	9	(9)					
No	137	24	(18)	2.00	0.97–4.13			
Unknown	427	80	(19)	2.14	1.11–4.13			

Unfavourable includes all outcomes other than cure or treatment completed; CI = Confidential interval; HIV =  Human immunodeficiency virus; DOT =  Directly observed treatment.

Multivariate analysis of the association of DM status with treatment outcome provided relative risk and corresponding confidence intervals, adjusted for age group, sex, site and type of TB, smear result and HIV status. Adjusted relative risk for unfavourable TB treatment outcome was statistically significant only for unknown DM status. Multivariate analysis could not be performed for regularity of DOT in IP nor for DM control status as the numbers of observations were too limited.

Table 4 gives the detailed outcome of all TB case stratified by DM status and DM control status during treatment. The highest proportion of unfavourable outcome was for default and death.

**Table 4:** Categories of Outcome of treatment of Tuberculosis in the Revised National Tuberculosis Control Programme in Malappuram District, Kerala, India, April 2010 to September 2011, by Diabetes Mellitus and Diabetes control status.

	Diabetes mellitus						Diabetes control status					
Outcome	Unknown		No		Yes		Unknown		Yes		No	
	n	(%)	n	(%)	n	(%)	n	(%)	n	(%)	n	(%)
All	322	(100)	2,127	(100)	677	(100)	427	(100)	103	(100)	137	(100)
Cured	109	(34)	899	(42)	383	(57)	240	(56)	61	(59)	82	(60)
Treatment Completed	140	(44)	940	(44)	171	(26)	107	(25)	33	(32)	31	(23)
Died	34	(11)	71	(3)	42	(6)	26	(6)	5	(5)	11	(8)
Failed	6	(2)	77	(4)	32	(5)	20	(5)	3	(3)	9	(7)
Defaulted	30	(11)	121	(6)	32	(7)	28	(7)	1	(1)	3	(2)
Transferred out	3	(1)	13	(1)	2	(1)	2	(1)	0	(0)	0	(0)
Switched to MDR-TB treatment	0	(0)	5	(0)	5	(1)	4	(1)	0	(0)	1	(1)

MDR TB = Multi drug resistant TB.

## Discussion

Our study did not confirm an adverse association between DM or DM control during treatment and the course of response to anti-tuberculosis treatment. This differs from other studies that have reported that tuberculosis patients with diabetes do not respond as well to the anti-tuberculosis treatment. One possible explanation for this difference is the fact that our study was a large study undertaken in a routine programme setting and including all tuberculosis patients in the peripheral health institutions in the study. We were also unable to precisely determine the adequacy of diabetes care because we did not have access to test glycosylated hemoglobin under programme condition for routine use. In addition we have no access to oral glucose tolerance test,which is the gold standard for diagnosis of diabetes mellitus,for routine use under the programme. Finally, the proportion of patients in whom the follow up of diabetes care was unknown was high. In a similar study from India by Dr.Hemalatha et al, evaluated the association between the poor glycemic control and severity of pulmonary TB.Thier conclusion was the uncontrolled diabetic patients are more prone for severe form of Pulmonary TB.In that study the study population was only 119 and they used both glycosylated hemoglobin and blood glucose estimation for analysis.

A major strength of our study was that it was conducted in a country with a high burden of tuberculosis and of diabetes mellitus. Moreover, it included all TB patients in the peripheral health facilities studied. It was a large study and unique in reporting the status of response to standard care for diabetic TB patients from a routine services setting.

Our study also had certain limitations. It was operational research and therefore dependent on routine records with their inherent errors and weakness. In addition the high proportion of patients in whom response to treatment of diabetes was unknown limits our confidence in the conclusions we can draw. We believe that this deficiency is largely related to the fact that standard care for diabetic TB patients in peripheral health institutions was at an early stage of implementation and therefore not as comprehensive as it could be [25]–[27].

Several actions are needed as a result of our findings. First we need to strengthen the recording of diabetes care in peripheral health institutions managing diabetic TB patients, in order to obtain more comprehensive information from which to draw evidence. The limitations of our study do not allow us to draw firm conclusions on the association between response to diabetes treatment and response to anti-tuberculosis treatment. Strengthening of routine recording is one of the steps to address this, but more robust study designs (such as prospective studies) would more likely to provide new knowledge on this issue.

We continue to face a high burden of both tuberculosis and of diabetes mellitus in our community. We need better information to guide us in caring for this ‘dual’ burden and we need to strengthen the care of these patients in our services. Co-ordinated management of diabetic care in association with National non communicable disease control programme and RNTCP is a promising opportunity.
